# Learning and memory formation in zebrafish: Protein dynamics and molecular tools

**DOI:** 10.3389/fcell.2023.1120984

**Published:** 2023-03-09

**Authors:** Kitty Reemst, Heba Shahin, Or David Shahar

**Affiliations:** ^1^ Migal—Galilee Research Institute, Kiryat Shmona, Israel; ^2^ Department of Biotechnology, Tel-Hai College, Kiryat Shmona, Israel

**Keywords:** zebrafish, memory consolidation, protein synthesis, memory, long-term memory, learning, translation

## Abstract

Research on learning and memory formation at the level of neural networks, as well as at the molecular level, is challenging due to the immense complexity of the brain. The zebrafish as a genetically tractable model organism can overcome many of the current challenges of studying molecular mechanisms of learning and memory formation. Zebrafish have a translucent, smaller and more accessible brain than that of mammals, allowing imaging of the entire brain during behavioral manipulations. Recent years have seen an extensive increase in published brain research describing the use of zebrafish for the study of learning and memory. Nevertheless, due to the complexity of the brain comprising many neural cell types that are difficult to isolate, it has been difficult to elucidate neural networks and molecular mechanisms involved in memory formation in an unbiased manner, even in zebrafish larvae. Therefore, data regarding the identity, location, and intensity of nascent proteins during memory formation is still sparse and our understanding of the molecular networks remains limited, indicating a need for new techniques. Here, we review recent progress in establishing learning paradigms for zebrafish and the development of methods to elucidate neural and molecular networks of learning. We describe various types of learning and highlight directions for future studies, focusing on molecular mechanisms of long-term memory formation and promising state-of-the-art techniques such as cell-type-specific metabolic labeling.

## Introduction

Learning and memory are an integral part of our daily lives. Cognitive and behavioral alterations during learning and memory are mediated by changes at the molecular level, most of which seem to be remarkably conserved across species (Kandel et al., 2014). Although research to understand the molecular mechanisms of learning and memory has made great progress, and several protein factors important for plasticity have been identified (Kelleher et al., 2004; Sutton and Schuman, 2006), the exact underlying molecular pathways are yet to be fully characterized (Evans et al., 2021).

**FIGURE 1:**
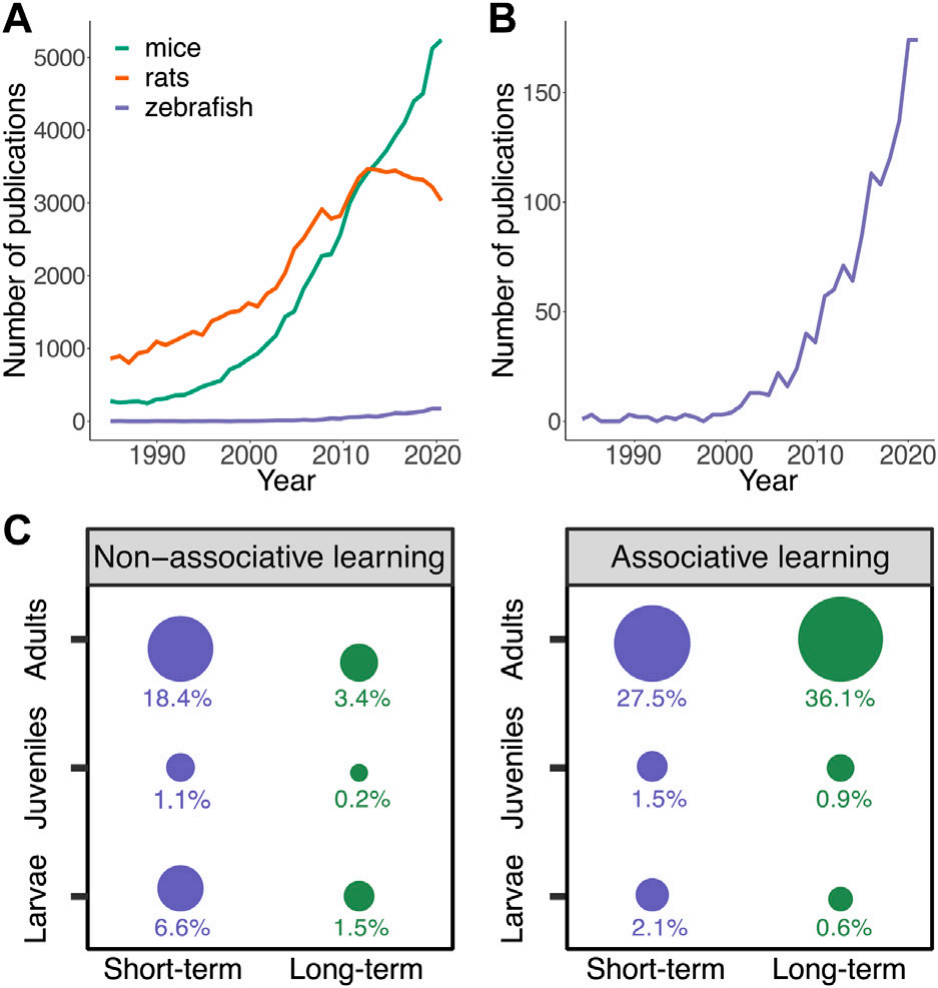
Studies of learning and memory formation in zebrafish. PubMed results for search terms (species; rats, mice or zebrafish) AND (learning OR memory). **(A)** PubMed results (1985–2021) for rats, mice and zebrafish indicating the dominance of rodents for studying learning and memory. **(B)** Focus on PubMed results for zebrafish indicating the increase in publications in zebrafish for studying learning and memory. **(C)** PubMed results (date of search 16/10/2022) for zebrafish were screened (N = 1,307) and 433 papers that we could relate to learning and memory formation (e.g., not including reviews or behavioral studies that we could not categorize as learning) were included and their data were classified for associative or non-associative learning, short- or long-term memory. Larvae were defined as younger than 13 dpf, juveniles as 13 dpf to 2 months old, and adults as 2 months or older. Size of the circle represents the percentage out of total included experiments. Data were analyzed and visualized using R.

A major obstacle to research focused on the neural networks and molecular pathways involved in learning and memory formation is the immense complexity of the brain. Therefore, efforts have been made to develop learning paradigms in model organisms with useful behavioral repertoires, including invertebrates with simple nervous systems and vertebrates in which overall regions of the central nervous system have been conserved. So far, most research on learning and memory has been performed in two highly related rodent model organisms, rats and mice (Figure 1A). The ability to form memories and recall previous events and thereby alter future behaviors provides such a strong evolutionary advantage that it is well conserved in both invertebrates and vertebrates including zebrafish (Farley and Alkon, 1985).

The zebrafish is a relative newcomer as a model organism in behavioral neuroscience, with an observed sharp increase in published studies on learning and memory in the recent decade (Figure 1B). Characteristics of zebrafish are beneficial to study the conserved and general principles of vertebrate nervous system functions, including learning and memory, making zebrafish a powerful model organism to study memory consolidation. Zebrafish are genetically tractable, translucent vertebrates with a small and relatively accessible brain that possesses numerous features of brain organization conserved across vertebrates. In addition, zebrafish exhibit complex behaviors, including learning and memory behaviors. Combining these characteristics with their suitability to high-throughput studies, zebrafish provides an excellent complementary vertebrate model for studying the molecular and neural basis of learning and memory (Gerlai, 2020).

Here, we provide a brief review of recent progress in learning paradigms for larval, juvenile, and adult zebrafish, as well as experimental methods to elucidate neural and molecular networks of learning and memory. We describe various types of learning and highlight directions for future studies, focusing on molecular mechanisms of long-term memory formation and promising state-of-the-art techniques, such as cell-type-specific metabolic labeling.

## Zebrafish as a model for studying learning and memory

Zebrafish has long been a favorite model organism for developmental biologists and geneticists, mainly due to their suitability for genetic manipulation and translucent nature from oocyte to developed larvae. Over the last 2 decades, researchers have begun to appreciate the organism’s usefulness for behavioral studies, including for social behavior and, more recently, for studying mechanisms of learning and memory formation. Here, we discuss 1) behaviors related to learning and memory formation, and 2) research paradigms used to study learning and memory in zebrafish.

### Zebrafish brain regions associated with learning and memory

Many basic neural systems mediating learning and memory have been evolutionarily conserved. Mammalian brain regions known to be key for memory, including the hippocampus and amygdala, have functional homologous structures in the zebrafish brain. The dorsomedial telencephalic region is involved in place preference conditioning as was demonstrated by activity of the immediately early gene *c-fos* (von Trotha et al., 2014). Further analyses of specific markers involved in emotional behaviors proposed this region to be homologous to the mammalian amygdala (von Trotha et al., 2014; Porter and Mueller, 2020; Lal et al., 2018). Likewise, the lateral pallium is involved in spatial learning and fulfills hippocampus-like functions (Broglio et al., 2010; Mueller, 2012; Mazzitelli-Fuentes et al., 2022).

In addition to studying established regions for memory consolidation, unbiased approaches of studying molecular mechanisms of learning and memory could identify additional conserved brain regions and networks important for the consolidation of memory, as discussed in the section “Zebrafish for the study of molecular mechanisms of learning and memory.”

### Behavioral repertoire related to learning and memory formation

Zebrafish exhibit a broad range of behaviors depending on their developmental stage (Nelson and Granato, 2022). These behaviors can be manipulated and studied in the context of learning and memory experiments. Within the first day post-fertilization (dpf), larvae exhibit spontaneous coiling (Granato and Nüsslein-Volhard, 1996; Saint-Amant and Drapeau, 1998). At three dpf, larvae react to touch by tail beating and backward movement and exhibit escape behavior when exposed to touch, acoustic, or electrical stimuli (Nelson and Granato, 2022; Santistevan et al., 2022). From three dpf, larval zebrafish avoid both hot and cold temperatures (Prober et al., 2008; Gau et al., 2013; Haesemeyer et al., 2015). At four dpf, larvae perform spontaneous swimming and start to exhibit habituation learning following repeated stimuli (Best et al., 2007). At five dpf, larvae can feed on their own, and at 6–7 dpf they actively hunt live prey, as well as keep a certain distance with respect to conspecifics (Marques et al., 2017). At 6–8 dpf, larvae show preference to a light over dark environment and actively swim to the light environment (Hinz et al., 2013). This light-dark preference will change to dark preference in adults, but the exact ontology is not yet known. Social preference starts 1 week post-fertilization and becomes robust at 3 weeks (Hinz et al., 2013; Dreosti et al., 2015). In accordance to the behavioral repertoire during development, numerous learning paradigms have been reported for zebrafish across ages (Kenney, 2020; R; Gerlai, 2016; Mu et al., 2020) (Figure 1C).

### Learning paradigms in zebrafish across ages

**FIGURE 2:**
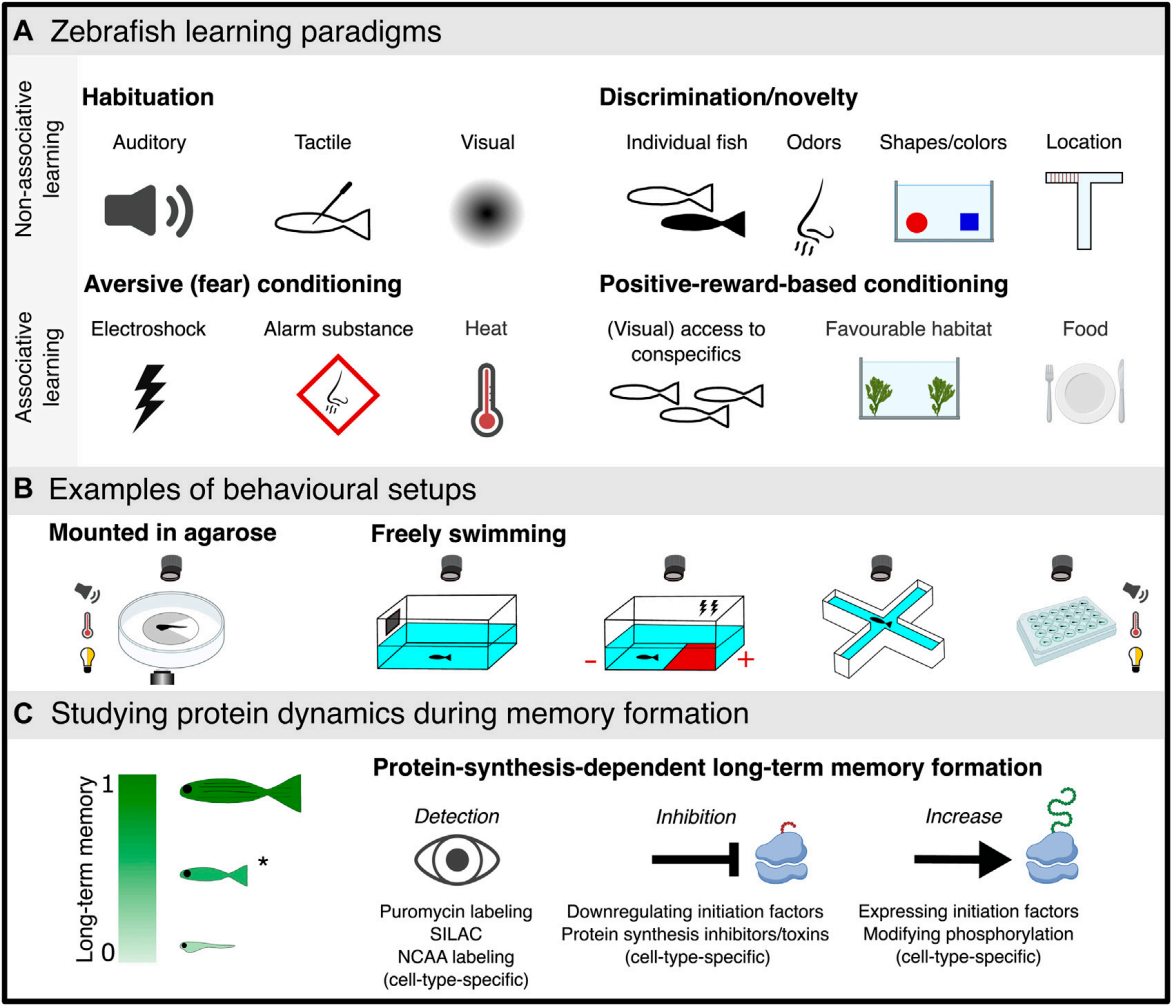
Schematic overview of studying learning in zebrafish. **(A)** Schematic representation of learning and memory paradigms used in zebrafish including forms of non-associative learning (habituation, recognition and novelty learning) and associative learning (aversive and positive-reward based learning). **(B)** Behavioural setups for zebrafish learning paradigms (partially mounted and freely swimming) can include recording of behaviour, neural activity combined with various stimuli as presented in **(A)**, or light-dark environment (white-yellow light bulb). **(C)** Protein dynamics during long-term memory formation. Left, the ability to form long-term memory increases with age (larvae, juvenile and adult from bottom to top, respectively) with only some evidence in larvae while most studies have used adult fish (Figure 1C), highlighting the rationale to further examine long-term memory consolidation in juvenile zebrafish (*) that possess a more accessible brain than adults. Right, a suite of methodologies allowing for the detection and manipulation of protein dynamics during protein-synthesis-dependent long-term memory formation. The figure was created with BioRender and Inkscape.

Larval zebrafish have been predominantly used to study habituation learning (Figure 2A) (paragraph 2. b.1; (Roberts et al., 2013), which has been demonstrated as early as 4 dpf (Eaton et al., 1977). In addition to this relatively simple form of learning, it was reported that 6–8 dpf larvae can perform in an associative place-preference paradigm (Hinz et al., 2013). At eight dpf, partially mounted larvae performed Relief of Aversive Stimulus by Turn (ROAST) in operant-conditioning task where the larvae could avoid an aversive heat stimulus by moving the tail (Figure 2B) (Lin et al., 2019). Simultaneous Ca^2+^ imaging revealed functional connectivity changes between the cerebellum and habenula, which correlated with decision outcomes. Other studies showed that ten dpf larvae are able to perform a visual lateralization novel object recognition task (Andersson et al., 2015). Classical and operant conditioning paradigms using electroshocks showed that learning improves during development from seven dpf, starts to be robust at around week three and reaches adult performance at week six (Valente et al., 2012). Another study demonstrated that a spatial alternation task based on a food reward was successfully performed by young adult (6–8 weeks) and adult fish (>8 weeks), while 3-4-week-old juveniles did not learn the task (Williams et al., 2002).

Adult zebrafish are able to perform complex learning tasks, which depends on their ability to discriminate between different sensory stimuli (Figure 2A). They can discriminate between visual stimuli such as shapes and colors (Colwill et al., 2005; Risner et al., 2005; Gatto et al., 2020; Santacà et al., 2021; Santaca et al., 2022), different odors (Braubach et al., 2008; Namekawa et al., 2018), and individual fish (Madeira and Oliveira, 2017). These cues have been used to train fish during complex spatial learning tasks (Williams et al., 2002; Levin et al., 2003; Xu et al., 2006; Baratti et al., 2019; Baratti et al., 2021). For example, fish can use the geometry of an arena to orient themselves in order to find the exit and gain a reward (Baratti et al., 2021).

Forms of avoidance and fear learning paradigms have also been widely used with zebrafish (Figure 2A) (Pradel et al., 2000; Xu et al., 2006; Castro et al., 2009; Baker and Wong, 2019). For example, adult zebrafish can form memories of a natural olfactory alarm cue using a contextual fear learning paradigm that depends on stress-coping styles of the zebrafish (Baker and Wong, 2019). Most associative learning paradigms resulting in long-term memory formation have been demonstrated in adult zebrafish (Figure 1C). A recent study used electroshock fear conditioning in juveniles and found that the dorsolateral habenula is required for updating learned behaviors (Palumbo et al., 2020). In addition to negative reinforcers or punishment, such as electric shocks and natural olfactory alarm cues, positive reinforcers have been studied for classical conditioning paradigms, including food (Bilotta et al., 2005; Colwill et al., 2005; Sison and Gerlai, 2009; Manabe et al., 2013a) and visual access to conspecifics (Al-Imari and Gerlai, 2008; Sison and Gerlai, 2011; Hinz et al., 2013; Fernandes et al., 2016). For example, adult zebrafish have been successfully trained to perform well during visual discrimination and amodal completion tasks, using both food and conspecifics as a reward (Sovrano et al., 2022).

#### Zebrafish paradigms for non-associative learning

Learning can be divided into two main forms: non-associative and associative (Figure 2A). Non-associative learning is a simple yet fundamental form of learning, not requiring stimuli association or pairing. It means that a response to a single event or stimulus, an animal can change their behavior (Ioannou and Anastassiou-Hadjicharalambous, 2021). Examples of non-associative learning include habituation, sensitization, perceptual learning, priming and recognition memory (Pereira and Kooy, 2013; Ioannou and Anastassiou-Hadjicharalambous, 2021). Habituation and sensitization learning are implicit or procedural forms of learning that respectively attenuates or augments (sensitizes) an animal’s sensory percept or behavioral response to a sensory stimulus upon repeated or continual presentation of the stimulus (Harris, 1943; Thompson and Spencer, 1966; Poon and Schmid, 2012). The altered response to the repeated stimuli of fixed intensity is not due to sensory adaptation, fatigue, or injury. Habituation has been commonly used in zebrafish paradigms based on a rapid startle response that decreases over time upon repeated exposure to a sensory stimulus (auditory, visual, or tactile) (Eaton et al., 1977; Best et al., 2007; Wolman et al., 2011). The light preference of larval zebrafish has been used to show dynamic learning including sensitization and habituation during a dark-avoidance task (Xu et al., 2021). The use of virtual reality paradigms with larval zebrafish embedded in agarose combined with light sheet microscopy allows for whole brain imaging during short-term motor learning (Kawashima et al., 2016). Regarding recognition learning, both short- and long-term memory formation have been demonstrated using novel object recognition and location paradigms in zebrafish (Lucon-Xiccato and Dadda, 2014; Oliveira et al., 2014; Andersson et al., 2015; May et al., 2015; Gaspary et al., 2018). Additionally, Y- and T-mazes have been primarily used to study working and short-term memory of a previously explored arm (Cleal et al., 2020; Fontana et al., 2021; Brinza et al., 2022).

#### Zebrafish paradigms for associative learning

Associative learning involves establishing a relationship (association) between at least two separate stimuli. A basic form of associative learning is classical conditioning (Pavlov, 1951; Sokolov, 1963; Rehman et al., 2022). Here, animals learn how to associate a neutral stimulus (conditioned stimulus, CS) with a reinforcing stimulus which can be either positive or negative (unconditioned stimulus, US) (Figure 2B). As the result of the paired delivery of a CS and US, the animal learns that the CS predicts the occurrence of the US. Consequently, the response to the CS becomes similar to its initial response to the US. Classical conditioning has been reported in both larval (Aizenberg and Schuman, 2011; Valente et al., 2012; Hinz et al., 2013) and adult zebrafish (Braubach et al., 2008; Agetsuma et al., 2010; Karnik and Gerlai, 2012; Aoki et al., 2013) using both positive (Colwill et al., 2005; Al-Imari and Gerlai, 2008; Gómez-Laplaza and Gerlai, 2009; Sison and Gerlai, 2009; Sison and Gerlai, 2011; Manabe et al., 2013b) and negative (Xu et al., 2006; Castro et al., 2009; Lee et al., 2010; Pradel et al., 2000; Levin and Chen, 2004; Baker and Wong, 2019) reinforcing stimuli. The use of classical conditioning in adult zebrafish is dominant in the literature (Figure 1C), yet, recent studies have adapted long-term memory associative learning-based paradigms for juveniles e.g., (Palumbo et al., 2020).

#### High-throughput paradigms

Zebrafish can breed throughout the year and have progenies of hundreds of eggs, which allows for studying memory formation in large numbers or while testing numerous conditions in a high-throughput manner. There have been developments in designing learning paradigms that are more appropriate for high-throughput screens (Stewart et al., 2015). For example, automated systems for imaging, tracking, and analyzing dozens of larvae simultaneously (Figure 1B) (Ahmed et al., 2010; Wolman et al., 2011; Doyle et al., 2016; Randlett et al., 2019; Barreiros et al., 2021); paradigms that can be performed with multiple adult fish at the same time (Kareklas et al., 2018; Samaras and Pavlidis, 2020; Barreiros et al., 2021); and relatively short paradigms that do not last for more than 2 or 3 days (Hinz et al., 2013; Lucon-Xiccato and Dadda, 2014). Efforts should be made to ensure that fish are habituated properly to the paradigm setup and show no signs of stress or anxiety, especially for paradigms that use individual zebrafish (Gerlai, 2016).

## Zebrafish for the study of molecular mechanisms of learning and memory

The zebrafish model is advantageous due its nervous system complexity and practical accessibility. Zebrafish are evolutionary ancient vertebrates but still possess numerous conserved features across multiple levels of biological organization in the brain. Zebrafish larvae are the only vertebrates with a translucent brain, allowing for imaging of the entire brain at a (sub)-cellular level, even while the fish is alive and during learning tasks described above (Naumann et al., 2016). Using optogenetic tools, it is possible to manipulate neuronal activity in a specific and reversible manner. Light-gated channels (Szobota et al., 2007; Douglass et al., 2008; Arrenberg et al., 2009; Bundschuh et al., 2012; Fajardo et al., 2013) can be used to either excite or inhibit neurons, which consequently can be imaged in the intact behaving fish (Wyart and Del Bene, 2011; Del Bene and Wyart, 2012; Portugues et al., 2013). The transparency of the larval zebrafish allows for non-invasive optogenetic detection and modulation of neural activity, and pharmacological tools and genetic lines exist that increase this transparency into adulthood (Karlsson et al., 2001; Bergmann et al., 2018). Indeed, there have been successful reports on the use of optogenetics to study both larval (Harmon et al., 2017) and adult zebrafish behavior (Douglass et al., 2008; Ahrens et al., 2012; Del Bene and Wyart, 2012; Portugues et al., 2013). Still, application during learning and memory paradigms is challenging, mainly due to the need for restraining the fish during the experimental procedure (Wyart et al., 2009).

### Protein-synthesis-dependent long-term memory formation in zebrafish

Both associative and non-associative learning can lead to short- or long-term memory formation. Short-term memory lasts from seconds to minutes and its formation mostly relies on biochemical changes to existing proteins (Kandel et al., 2014). Long-term memory lasts from hours to years and its formation is protein synthesis dependent (Flexner et al., 1962; Josefa et al., 1963; Nee et al., 2008). This has been demonstrated in different organisms and using various learning paradigms (Agranoff et al., 1966; Gal-Ben-Ari and Rosenblum, 2011; Gal-Ben-Ari et al., 2012; Kandel et al., 2014).

In zebrafish, both long-term habituation (Wolman et al., 2011; Roberts et al., 2013; Roberts et al., 2016) and classical conditioning leading to long term memory formation (Pradel et al., 1998; Pradel et al., 2000; Blank et al., 2009) depend on the ability to synthesize new proteins. For example, an essential role for the synthesis of cell adhesion molecules in memory consolidation and recall in adult zebrafish was discovered using an active avoidance paradigm (Pradel et al., 2000). A conditioned place preference paradigm in zebrafish larvae reported protein-synthesis-dependent long-term memory formation and a role for NMDA-receptor activation in this process (Hinz et al., 2013). The place preference for an environment with visual access to conspecifics develops with age and becomes more robust 2–3 weeks post fertilization (Dreosti et al., 2015), suggesting that this promising paradigm may be more robust in juvenile zebrafish (Figure 2C). A recent study measured brain protein dynamics following adaptation of zebrafish to water currents induced by magnetic stirrers and detected 57 regulated proteins in larvae exposed to the water vortex (Langebeck-Jensen et al., 2019). However, when measuring total protein content, newly synthesized (nascent) proteins can be masked by already existing proteins, thus hindering their detection. In addition, the use of whole tissue proteomics hinders detection of cell-type-specific protein alterations. This calls for methodologies that allow for the detection of newly synthesized proteins, preferably in cell types of interest.

### Manipulation of protein synthesis

Due to the complexity of the brain, comprising many cell types including neurons that possess long processes and are entangled in the respective tissue, it is difficult to reveal the newly synthesized proteome during learning and memory formation in an unbiased manner. In this section, we will review evidence for the role of protein synthesis during long-term memory formation and discuss novel methods that allow for the labelling of nascent proteins in cell types of interest (Figure 1C). See also reviews on *de novo* proteomic methods in relation to memory consolidation (Evans et al., 2021; Ross et al., 2021).

The pioneering studies that first showed the need for protein synthesis during long-term memory formation used protein synthesis inhibitors such as puromycin, delivered non-specifically (Flexner et al., 1963). Delivering protein synthesis inhibitors can done by injection to a brain region or for zebrafish, by adding it to the water bath of the fish resulting in universal inhibition (Hinz et al., 2012; Shahar and Schuman, 2020). However, within the complex structure of the brain, different brain regions and cell types enable the formation, consolidation, and recall of memory (Camina and Güell, 2017). Although inhibitors can be delivered directly to specific brain regions at different stages of long-term memory formation, they cannot be restricted to specific cell types and therefore cannot distinguish between the role of neuronal subtypes or glial cells in memory consolidation. Furthermore, although memory research has predominantly focused on hippocampal neurons, other brain cells including glia have been shown to play a role too (Yoo et al., 2021). Recently, tools that enable cell-type-specific drug-inducible inhibition of protein synthesis have been developed including a toxin from Maize that can be expressed in a cell-type-specific manner (Heumüller et al., 2019) which has so far only been demonstrated *in vitro*. Another approach enables rapid and reversible phosphorylation of eukaryotic initiation factor 2α, leading to inhibition of general translation (Shrestha et al., 2020). Such tools have the potential to increase the spatiotemporal resolution in which protein synthesis can be detected during learning and memory adaptations. Similarly, protein synthesis has been artificially increased in specific cell types through overexpression of initiation factors (Shrestha et al., 2020; Xu et al., 2020). Another way to modulate translation of memory consolidation related proteins and in a cell-type-specific manner is by modifying the kinase activity of elf2a, which affects memory consolidation in mice (Gould et al., 2020; Sharma et al., 2020). Future studies in zebrafish could provide additional information on conserved vertebrate brain regions and cell types involved in different stages of memory formation.

### Labeling of nascent proteins

Given the evidence that protein synthesis is required for long-term memory formation, identifying which proteins are synthesized, and their specific roles, is fundamental for understanding the complexity of the underpinning molecular mechanisms (Flexner et al., 1963; Sutton and Schuman, 2006; Costa-Mattioli et al., 2008; Gal-Ben-Ari et al., 2012; Hinz et al., 2013). Manipulation of protein synthesis provides useful information about the importance of protein synthesis in certain cell types and brain regions during memory consolidation, but it does not identify which proteins are being synthesized. Therefore, efforts have been made to identify newly synthesized proteins in a given brain region in a cell-type-specific manner. One widely used technique is Stable Isotope Labelling with Amino Acids in cell culture (SILAC) (Koren et al., 2019). Isotope tagging of proteins leads to a shift in the molecular mass of the labelled peptide, thus enabling identification *via* mass spectrometry (Engmann et al., 2010; Chen et al., 2015). However, although this technique labels the newly synthesized proteins, it does not enrich for them, therefore, newly synthesized proteins in low abundance may be missed due to masking by highly abundant existing proteins. Additionally, although the technique has been adapted for its use in animal models (Price et al., 2010; Rauniyar et al., 2013), it cannot be directed to specific cell types, since it uses endogenous amino acids to label newly synthesized proteins.

A method that purifies only nascent proteins uses the general protein synthesis inhibitor puromycin (Nemoto et al., 1999). Puromycin is molecularly similar to aminoacyl-tRNA and uses the endogenous translational machinery to integrate itself into proteins as they are being synthesized (Dieck et al., 2015). Puromycin-tagged proteins can be labeled with anti-puromycin antibodies and subsequently visualized *via* immunohistochemistry, or purified and identified *via* mass spectrometry. Because of the rapid integration of puromycin into the newly synthesized amino-acid chain, this method can be used to examine local protein synthesis (Hafner et al., 2019). This is important because both in neurons and glial cells, it has been demonstrated that protein synthesis occurs locally, which likely plays an important role in synaptic plasticity (Sutton and Schuman, 2006; Sakers et al., 2017; Spaulding and Burgess, 2017). Click-chemistry compatible analogs of puromycin have been developed (Liu et al., 2012) and used to label neuronal nascent proteins (Holt et al., 2019). A disadvantage of puromycilation is its interference with the translation machinery and the resulting truncated peptides (Schmidt et al., 2009) and recent evidence suggests that puromycilation may not be a good indicator of nascent proteins (Enam et al., 2020; Hobson et al., 2020). Puromycin-independent techniques have been developed as well.

Non-canonical amino acid tagging (NCAT) has emerged as a strategy for identifying nascent proteins without terminating translation. Bio-Orthogonal NCAT (BONCAT) and Fluorescent NCAT (FUNCAT) methodologies tag newly synthesized proteins with either azide- or alkyne-bearing non-canonical amino acids (NCAAs) (Kiick et al., 2002; Link et al., 2004; Yang et al., 2018). Using click chemistry, the azide or alkyne group of the NCAA can be clicked to biotin for purification and mass spectrometry analysis (BONCAT), or a fluorophore for imaging (FUNCAT) (Hinz et al., 2012; Ngo et al., 2013; Ullrich et al., 2014; Lehner et al., 2017). As a result of the low toxicity, higher concentrations of NCAAs and longer labelling periods can be used *in vivo*, increasing proteome coverage (Koren et al., 2019). Moreover, since NCAAs do not affect the rate or efficiency of protein translation (Calve et al., 2016), this method is much more suitable for examining protein synthesis during long-term memory formation.

### Cell-type-specific labeling of nascent proteins

Modifications to NCAAs allow for cell-type-specific tagging of newly synthesized proteins. For example, the NCAA azidonorleucine (ANL) is not recognized by the endogenous methionine tRNA synthetase and therefore does not integrate into proteins in wild-type cells. ANL incorporates only into newly synthesized proteins in cells expressing mutant methionyl-tRNA synthetase (MetRS) in which, in zebrafish, Leucine 270 is replaced with Glycine (MetRS^L270G^) (Shahar and Schuman, 2020). In mice, this technology has been successfully used to label nascent proteins in hippocampal excitatory principal neurons and cerebellar Purkinje neurons, to discover differentially regulated proteins in mice exposed to an enriched environment (Alvarez-Castelao et al., 2017) and to identify 156 proteins in hippocampal excitatory neurons during an aversive cue learning paradigm (Evans et al., 2020). We have used both BONCAT and FUNCAT in zebrafish larvae expressing cell-type-specific MetRS^L270G^ in a pan-neuronal manner to label neuron-specific nascent protein, which revealed elevated levels of neuronal newly synthesized proteins following induced neuronal activity (Shahar and Schuman, 2020). FUNCAT provides spatial information and indicates the intensity of newly synthesized proteins. Moreover, a Proximity Ligation Assay (PLA) can be used in combination with FUNCAT (PLA-FUNCAT) to reveal the cellular location of nascent proteins-of-interest (Dieck et al., 2015; Evans et al., 2019). The PLA detects the spatial colocalization of two antibodies: one that identifies the newly synthesized protein by using click chemistry to the azide or alkyne group of the NCAA, and another that identifies a specific epitope in a protein of interest. Only when the two antibodies are in proximity, will a ligation amplification circle reaction occur, resulting in fluorescent signal (Dieck et al., 2015). The relative ease of creating transgenic zebrafish lines expressing mutant MetRS under glial and neuronal subtype promotors enables the exciting opportunity to identify cell-type-specific protein synthesis during long-term memory formation in vertebrates.

Thus, NCAT-based methods seem very promising for the analysis of protein-synthesis-dependent memory formation. It is important, however, to realize that strategies for NCAA delivery and labeling durations can affect experimental outcomes, which likely differs between species. While longer labelling periods enable the labeling of a large number of newly synthesized proteins, shorter labelling periods may be preferable for examining memory-phase-specific protein synthesis. In addition, labeling tools for nascent proteins have a bias towards proteins with a high turnover, because they are synthesized more regularly and thus more likely integrate the NCAA or puromycin (Schanzenbacher et al., 2016). Lastly, while these methods are able to identify the nascent proteome, it does not reveal their functionality. Their identity can be used for functional prediction, which can be further examined as next steps.

## Conclusion

The characteristics of zebrafish, including their 1) ability to learn, 2) relative ease of genetic manipulation, 3) suitability for high-throughput studies, and 4) translucent brain in young ages, make it an excellent vertebrate model to study the molecular underpinnings of learning and memory. The collective data thus far suggest that larval stages are easier to work with due to the more accessible brain and translucency, and that complex learning such as long-term memory formation work better in juveniles, starting at about two to 3 weeks post-fertilization and becoming more robust in adults. The fact that the brain of young juveniles (2–4 weeks post fertilization) is smaller and more accessible than that of adult zebrafish, poises them as a promising tool for future research. Combining young juveniles with advanced, novel techniques, in particular labeling with NCAAs in a cell-type-specific manner, is relatively unexplored but starting to be used for examining the nascent proteome during various forms of learning and memory. We envision a rise in the use and further refinement of the zebrafish model, including applications of these novel techniques, which will hopefully increase the understanding of conserved mechanisms of long-term memory formation in vertebrates at the molecular level.
